# High-throughput clinical antimicrobial susceptibility testing and drug-resistant subpopulation detection in Gram-negative bacteria

**DOI:** 10.1128/spectrum.00011-25

**Published:** 2025-06-05

**Authors:** QingFeng Hu, Yuhan Zhou, Yongze Zhu, Zixuan Ma, Tongtong Li, Shuhang Yao, Ju Pan, Mengyan Shi, Fang Su, Beiqiong Shen, Xi Li, Benfang Helen Ruan, Jiaxue Wang

**Affiliations:** 1 School of Laboratory Medicine and Bioengineering, Hangzhou Medical College 117839, Hangzhou, Zhejiang, China; 2 Zhejiang Provincial People's Hospital (Affiliated People's Hospital), Hangzhou Medical College 117839, Hangzhou, Zhejiang, China; 3 College of Pharmaceutical Science, Institute of Drug Development and Chemical Biology, Zhejiang University of Technology 630537, Hangzhou, Zhejiang, China; The University of North Carolina at Chapel Hill, Chapel Hill, North Carolina, USA

**Keywords:** Gram-negative bacteria, antimicrobial susceptibility testing, heteroresistance, AST, drug-resistant bacterial subpopulation, EZMTT

## Abstract

The optimized Easy and Zooming MTT (EZMTT)-based antimicrobial susceptibility testing (AST) method for Gram-negative bacteria demonstrated 5- to 20-fold enhanced sensitivity and improved the detection limit to approximately 1.13% ± 0.30% growth. This advancement enables the detection of drug-resistant subpopulations that were previously undetectable by the gold standard broth microdilution (BMD) method, which has a growth limitation of approximately 10.15% ± 4.70%. Consistent results were achieved through AST of 205 clinical bacterial samples using both the EZMTT and BMD methods. At 450 nm, the overall positive and negative compliance rates were 98.94% and 99.58%, respectively, with a kappa value of 0.981 and a 95% CI of 0.974–0.988. For each of the 23 antibiotics tested, compliance rates exceeded 90.00%, with kappa values above 0.794 ~ 1.000 for 21 of them. Notably, the AST screening identified a clinical *K. pneumoniae* strain (KP007) containing drug-resistant subpopulations. Further analysis using the K-B method identified 13 drug-resistant subpopulations in KP007, with detection rates of 100% by the EZMTT method, 77% by the BMD method, and less than 44% by the VITEK method. A series of heteroresistant strains was generated by mixing the parent-sensitive strain (DH10B) with fivefold dilutions of its resistant strain transformed with an ampicillin resistance gene (DH10B-Amp^R^). Results indicated that the EZMTT method is more than 3,000 times more effective at detecting the resistant subpopulation (DH10B-Amp^R^) compared to VITEK. In conclusion, this study demonstrates that the EZMTT method exhibits good consistency and stability and is a highly sensitive AST method for detecting drug-resistant subpopulations in Gram-negative bacteria.

**IMPORTANCE:**

Bacterial drug resistance represents a significant global health concern. Currently, the minimum inhibitory concentration (MIC) is widely accepted as a primary criterion for determining bacterial sensitivity or resistance. However, pathogenic bacteria classified as sensitive based on MIC criteria may still exhibit resistance. One crucial factor contributing to this phenomenon is the heterogeneity of bacterial resistance. Due to the small number of drug-resistant bacteria in these subpopulations, their corresponding drug resistance is often challenging to detect in MIC data. AST remains a critical method for guiding the use of antibacterial agents. In this study, we employed the EZMTT method to accurately detect drug-resistant bacterial subpopulations. This method significantly improved assay consistency, stability, and sensitivity in clinical bacterial strains when compared with other methods. Consequently, the application of EZMTT-modified BMD assay in clinical settings may potentially prevent the development of multidrug-resistant bacteria and enhance the treatment of infectious diseases in the future.

## INTRODUCTION

The issue of multidrug-resistant bacteria had become widespread by the time the World Health Organization (WHO) released its first global antibiotic resistance report in 2014. Bacterial resistance not only extends patients’ illness duration but also increases healthcare costs and presents a significant public health threat (1, 2). A 2020 study by the Chinese Antimicrobial Resistance Surveillance System revealed that the average bacterial resistance rate to certain antibacterial agents currently ranges from 18% to 53%. The primary antimicrobial susceptibility testing (AST) methods utilized in clinical microbiology laboratories include agar dilution, broth dilution, disk diffusion, gradient diffusion, and automated instrumentation (3, 4). The minimum inhibitory concentration (MIC), which is recommended by the United States Clinical and Laboratory Standards Institute (CLSI), is recorded as the measure of antibiotic-induced growth inhibition. In the disk diffusion method, a drug-loaded paper disk is placed on a bacterium-inoculated agar plate; after incubation, inhibited bacteria form a transparent circle of sterile agar, known as an inhibition zone (5). This method’s advantages include low cost and ease of interpretation, while its disadvantages include a lack of precision, low throughput, and susceptibility to human factors. The antibacterial agent concentration gradient method, such as the E-test, employs test strips marked with antimicrobial drug concentrations, allowing for easy and accurate MIC determination (6). However, these test strips are relatively expensive. Automated instrumentation, the fourth type of AST, includes systems like VITEK (7) and MicroScan microbial identification (8). Automation offers simplified procedures, and the turbidity-based method can be rapidly implemented (8–12 hours) using 10-fold more concentrated bacterial cultures. These AST methods have been widely employed in hospitals for over a decade. Despite this, the prevalence of drug-resistant or multidrug-resistant bacteria continues to increase annually (9, 10).

Recently, heterogeneous bacterial drug resistance has been identified as a distinct form of resistance, representing an intermediate stage in the evolution of bacterial drug resistance (drug-resistant subpopulations) (11). In bacterial communities, the presence of drug-resistant bacterial subpopulations is significantly associated with multidrug resistance (12). Unfortunately, due to the 20% growth detection limitation, many drug-resistant subpopulations with less than 20% growth remain undetected by current routine clinical ASTs; the resulting “sensitive” report may mislead physicians in prescribing, potentially leading to recurrent infections and therapeutic failure (13). Moreover, traditional methods for detecting drug sensitivity often require extended periods, which can result in missed diagnoses, misdiagnoses, and delayed treatment (14–16). Consequently, there is an urgent need for a new method with a substantially improved detection limit to identify both primary and minor drug-resistant bacterial subpopulations.

EZMTT, a novel monosulfonic acid tetrazolium salt (2-(3-(2- methoxy-4-nitrophenyl)−2-(4-nitrophenyl)−2H-tetrazol-3-ium-5-yl) benzenesulfonate sodium salt), was developed by our research group. Under optimized assay conditions, this compound demonstrates non-toxicity (17–19), stability, and rapid reactivity with NAD(P)H in living cells, producing a soluble orange–yellow color (OD_450nm_) (18); EZMTT significantly enhances the single living cell turbidity signal by detecting its thousands of cellular cofactor NAD(P)H, thereby improving the detection limit to less than 5% growth and facilitating the discovery of additional “hidden” drug-resistant bacterial subpopulations (20).

This study presents the AST evaluations and clinical trial data of Gram-negative bacteria using both the EZMTT-based and the broth microdilution (BMD) methods. The optimized EZMTT method demonstrated high precision, sensitivity, and reproducibility. The clinical trial results show strong concordance between the two methods when analyzed using the CLSI breakpoint standards. Furthermore, this study successfully identified strains containing minor drug-resistant bacterial subpopulations that were not detected by either the BMD or the VITEK methods. Collectively, these findings suggest that the EZMTT-based AST method holds significant potential for development into a valuable assay with broad future applications.

## MATERIALS AND METHODS

### Sample collection

**Fig 1:**
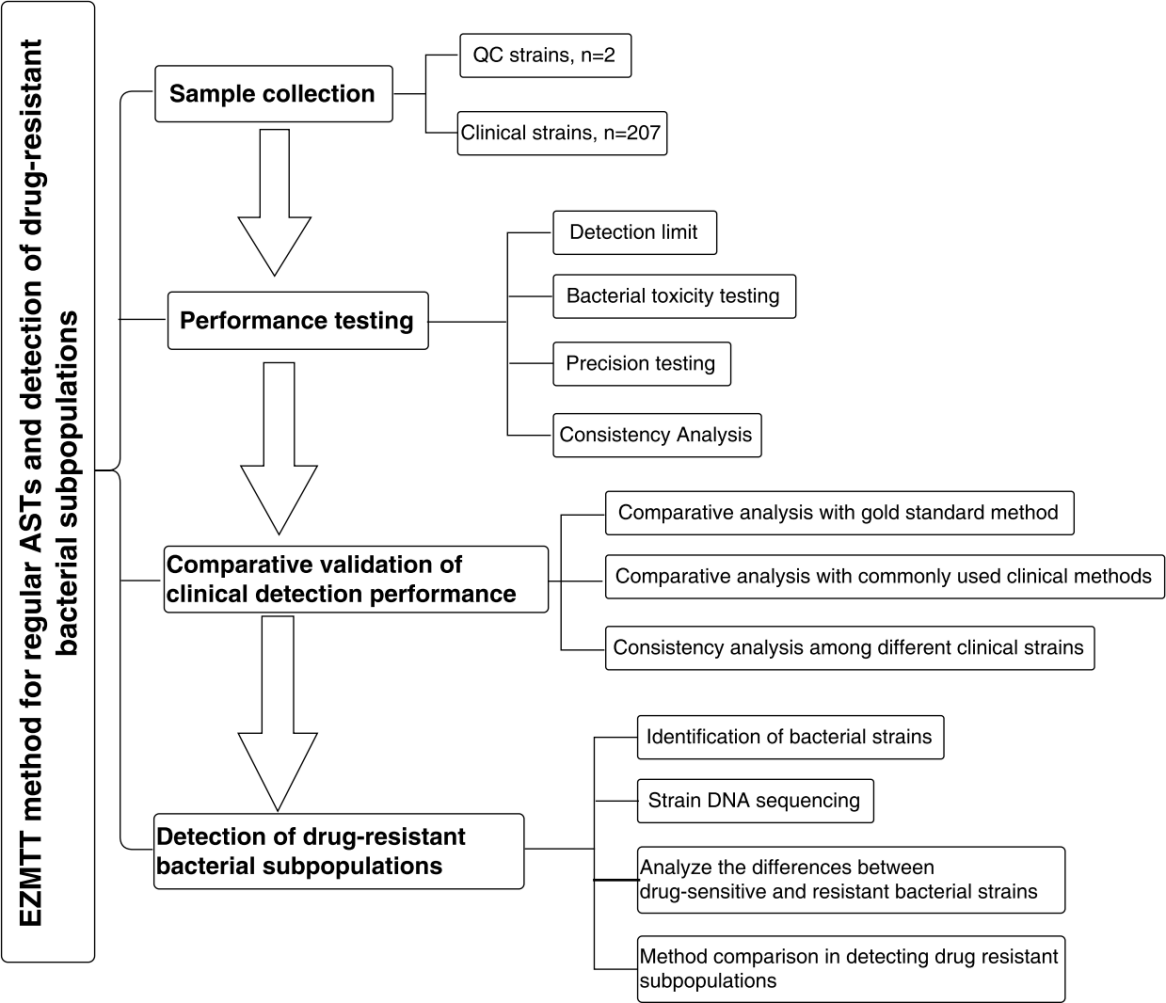
Flowchart (Fig. 1) of the study design.

From July 31, 2021, to August 24, 2021, a total of 207 samples of various types were identified as specific Gram-negative strains using matrix-assisted laser desorption ionization flight mass spectrometry: *E. coli, K. pneumoniae, A. baumannii,* and *P. aeruginosa*. The samples were obtained from Run Run Shaw Hospital of Zhejiang University School of Medicine and Zhejiang Provincial People’s Hospital. The *E. coli* strain ATCC 25922 and *Pseudomonas aeruginosa* strain ATCC 27853 served as internal quality controls to evaluate the accuracy and sensitivity of the EZMTT method. In addition, six more Gram-negative bacterial species such as *Enterobacter cloacae, Klebsiella pneumoniae, Citrobacter SPP., Proteus SPP, Acinetobacter baumannii,* and *Stenotrophomonas maltophilia* were examined. The quality control bacterial strains utilized in this study were acquired from Shanghai Preservation Biotechnology Center. The overall flowchart of this study design is shown in Fig. 1 .

### Materials

Ciprofloxacin (CIP), cefazolin (CZO), nitrofurantoin (NIT), meropenem (MEM), imipenem (IPM), cefuroxime (CXM), cefoperazone sulbactam (CSL), tigecycline (TGC), ampicillin (AMP), cefoxitin (FOX), tobramycin (TOB), minocycline (MNO), polymyxin (POL), trimethoprim/sulphamethoxazole (SXT), ceftazidime (CAZ), gentamicin (GEN), aztreonam (ATM), amikacin (AMK), amoxicillin-clavulanic acid (AMC), levofloxacin (LVX), ceftriaxone (CRO), cefepime (FEP), and piperacillin/tazobactam (TZP) were acquired from Solarbio LLC (Beijing, China) or Kangtai LLC (Wenzhou, China). The EZMTT reagents, automatic liquid dispenser, and the AST data processing software were purchased from Hangzhou Hanjing Bioscience LLC (Hangzhou, China). The VITEK 2 compact automatic microbiology instrument was obtained from Merckux LLC (France). Kirby-Bauer (K-B) papers impregnated with the drug were procured from Thermoﬁsher (USA). DNA sequencing was conducted by Zhejiang Tianke High Tech Development Co., Ltd.

### EZMTT assay procedure

In accordance with the CLSI standard drug sensitivity method, the minimum inhibitory concentrations (MICs) of various antimicrobial drugs for Gram-negative bacteria were determined using the broth microdilution (BMD) method in the presence or absence of the EZMTT reagents. A total of 23 formulated drugs were loaded onto two types of dilution plates, as shown in Fig. S1; GN#1, GN#2, and GN#3 plates are the control plates that are loaded with the 23 drugs in twofold dilutions. The GN plate is the testing plate loaded with 23 drugs, and their concentration selection was based on the known CLSI breakpoints for Gram-negative bacteria. The procedure was as follows: Fresh colonies cultured for 18–24 h were collected with an inoculating loop, added to sterile saline, aspirated 10 times with a 1 mL pipet, vortexed for 1 min, and then turbidimetrically measured to achieve a 0.5 McFarland dilution, which was to be used within 20 min. The 0.5 McFarland bacterial solution was diluted at a ratio (1:200) in cation-adjusted Mueller-Hinton broth (CAMHB) medium containing the EZMTT indicator (1 × final) and mixed thoroughly. The resulting bacterial mixture (100 µL) was added using an automatic liquid dispenser to all wells of a 96-well plate except for the 12G and 12H wells, which were loaded with EZMTT-broth only as a negative control. After being shaken at room temperature for 10‒20 minutes (at 100 rpm), the plates were incubated at 37°C for 20 hours (or overnight), and the AST results were measured via visual inspection and a plate reader, followed by one-click automatic data analysis. When the positive control exhibits a reddish-brown or orange‒yellow color and the negative control is light yellow or colorless, it initially indicates that bacterial growth is normal, the reagents are not contaminated, and the procedure has been performed correctly.

### Criteria for the EZMTT assay and data processing

A microplate reader measured the absorbance at 450 nm, and the growth rate for each well was calculated using the “Drug-plate” software from Hangzhou Hanjing Bioscience LLC (Hangzhou, China). The growth rate of the drug was assessed from low to high concentrations, assuming saturated growth in positive control wells. The MIC value was determined by the first non-growth well with a growth rate below 30%. If all wells containing the same drug exhibited a growth rate exceeding 70%, the MIC value was considered greater than or equal to the highest drug concentration; conversely, if all wells showed a growth rate below 30%, the MIC value was deemed less than the lowest drug concentration. Visual inspection could also be employed for data analysis. In the EZMTT method, orange–red coloration indicates growth, and the MIC value is determined by the concentration in the first well lacking this color. If the MIC value falls on or above the upper concentration of the breakpoint, the strain is classified as resistant (R) to the corresponding antibacterial agent. If the MIC value falls on or below the lower concentration of the breakpoint, the strain is classified as sensitive (S). If the MIC value falls in between the lower and the upper concentrations of the breakpoints, the strain is classified as intermediate (I).

### Rapid microbial mass spectrometry and whole-genome DNA sequencing

A single bacterial colony was selected using an inoculation loop and transferred directly to the designated sample spot on the VITEK MS-DS target plate, without any agar medium. Immediately after applying the bacteria, VITEK MS-CHCA substrate solution was added to the spot to facilitate cell lysis. Once dried, the target plate was placed into the VITEK MS loading bin for automated bacterial identification. Concurrently, bacterial colonies were collected separately for whole-genome sequencing, which was performed using both a NovaSeq 6000 sequencer and a GridION X5 sequencer. The resulting genome sequences were analyzed for antibiotic resistance genes using the Comprehensive Antibiotic Resistance Database (CARD).

### KB method

The K-B method adhered strictly to the procedure recommended by the CLSI. In brief, a single colony was selected and inoculated into 0.5 McFarland in saline. The bacterial suspension was then collected using a sterile cotton swab and uniformly spread on the surface of a solid agar plate. The antibiotic-impregnated disc was gently pressed onto the agar surface to ensure complete contact. Following incubation at 37°C for 16–18 hours, the diameter of the inhibition zone and any colony growth within this zone were assessed in accordance with CLSI standards.

### Statistical analysis

All experiments were conducted in triplicate and independently replicated three times. Quantitative data are presented as means ± standard deviations (SDs). Data analysis and visualization were performed using Origin 2022 statistical software and GraphPad Prism 9.5. For comparisons between two groups, *P* values were calculated using Student’s *t* test. *P < 0.05; **P < 0.01; ***P < 0.001; ****P < 0.0001. In all comparisons of categorical variables, *P <* 0.05 was deemed statistically significant.

## RESULTS

### Sample collection and analysis

In accordance with the Technical Guidelines for Clinical Trials of *In Vitro* Diagnostic Reagents, the total sample size for clinical trials of Class II products should be at least 200. Consequently, for clinical validation, 50 cases were allocated to each of the top four Gram-negative bacteria, with carbapenem-resistant strains comprising more than 30% (≥ 60) of the total sample size. Initially, 215 samples were enrolled, with eight subsequently excluded. Thus, the final number of samples included in the Full Analysis Set (FAS) and Per-Protocol Set (PPS) analysis was 207. The study encompassed four types of gram-negative bacteria: 56 cases of *E. coli* (27.0%), 50 cases of *K. pneumoniae* (24.15%), 52 cases of *A. baumannii* (25.12%), and 49 cases of *P. aeruginosa* (23.67%). Of these, 114 were identified as carbapenem-resistant bacteria, which consisted of over 30% of the carbapenem-resistant bacteria within each of the four types of gram-negative bacteria studied. The clinical strains were obtained from the medical laboratory centers of Run Run Shaw Hospital affiliated with Zhejiang University and Zhejiang Provincial People’s Hospital. Detailed information is presented in Table 1.

**TABLE 1:** Detailed distribution of samples used in the current study (FAS/PPS set)^*a*^

Index	A sample number (n)	B sample number (n)	Total sample number (n)	Proportion to total sample size (%）
Types of bacterial strains				
*K. pneumoniae*	25	25	50	24.15
*E. coli*	26	30	56	27.05
*P. aeruginosa*	25	24	49	23.67
*A. baumannii*	25	27	52	25.12
Total	101	106	207	100.00
Sample source				
Blood	32	13	45	21.74
Urine	14	22	36	17.39
Sputum	39	48	87	42.03
Body fluid	13	11	24	11.59
Other	3	12	15	7.25
Total	101	106	207	100.00
Drug sensitivity characteristics				
Drug-resistant bacterial strains	50	64	114	55.07
Drug-sensitive strains	51	42	93	44.93
Total	101	106	207	00

^*a*^ The EZMTT method was used to detect the same sample as the control group, and the sample distribution was the same. A: Samples were from the Medical Laboratory Center of Run Run Shaw Hospital, Zhejiang University. B: Samples were obtained from the Medical Laboratory Center of Zhejiang Provincial People's Hospital.

### The EZMTT-based method demonstrates a high signal value and a low detection threshold

**Fig 2:**
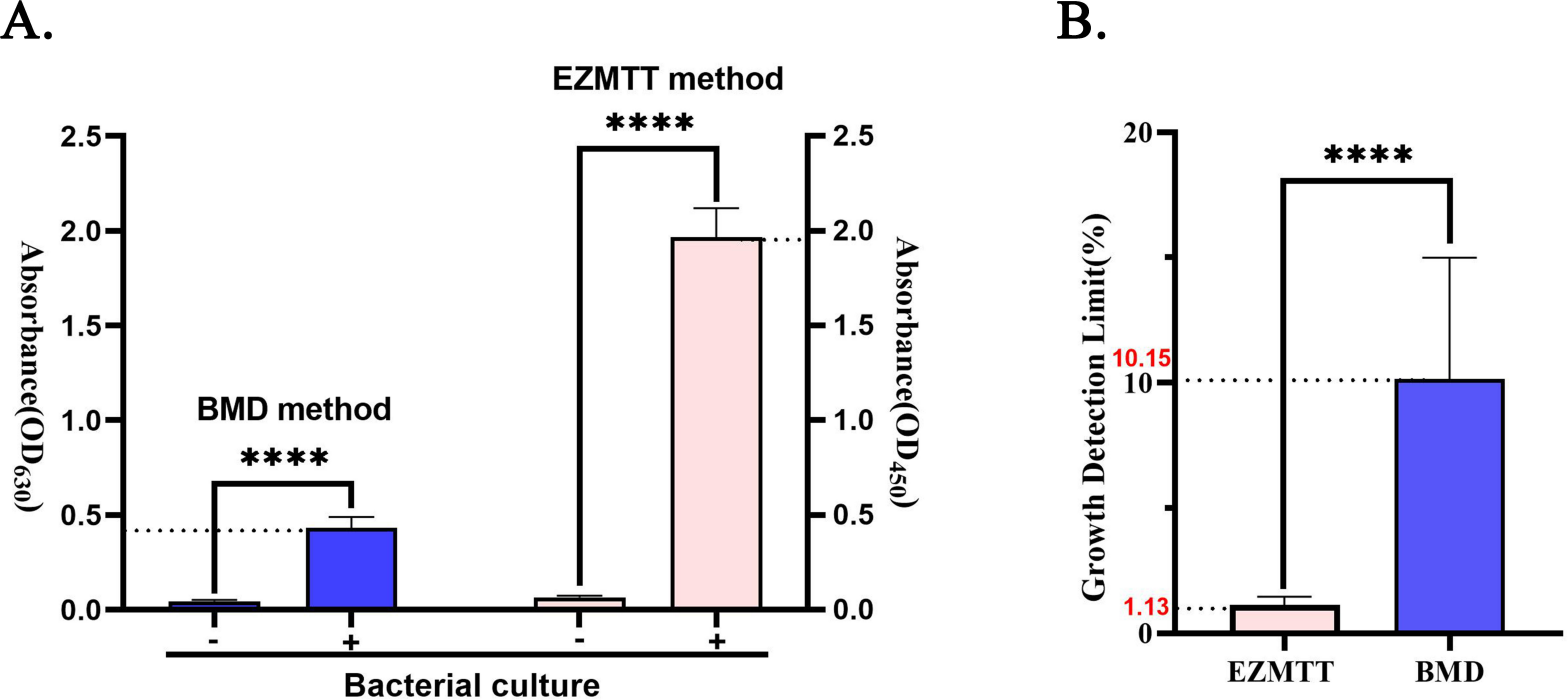
Signal, background, and growth detection limit for the EZMTT-based and the BMD methods. (**A**) Absorbance of the blank and the growth signal were determined via the EZMTT (OD_450_ nm) and the BMD methods (OD_630_ nm). (**B**) Growth detection limit of the EZMTT-based and the BMD methods, with threefold variation divided by the bacterial culture signal. Statistical analysis revealed a dramatic decrease of approximately 10-fold in the threshold for the EZMTT method, with a *P* value of < 0.0001 (****).

The background signal values and detection limits were evaluated using the BMD method in the presence or absence of EZMTT. The absorbance at 450 nm represents the growth signal generated by EZMTT, while the absorbance at 630 nm measures the turbidity of bacterial growth. Absorbance at both wavelengths was measured for blank 96-well plates and plates inoculated with bacterial culture (ATCC25922) for 24 hours with or without EZMTT. Figure 2 illustrates that the average absorbance of bacterial cultures was 1.932 and 0.453 in the presence and absence of EZMTT, respectively (Fig. 2A). The detection limits for both methods were calculated using the signal-to-noise ratio, defined as the background OD value plus threefold variation. The growth detection limit is determined by timing threefold variation of the background growth signal (OD_blank_ × CV × 3/(OD_positive_-OD_blank_) (21); the resulting growth detection limits were approximately 1.13% ± 0.30% and 10.15% ± 4.70% for the EZMTT-based and BMD methods, respectively (Fig. 2B). Consequently, EZMTT addition enhanced the detection limit by nearly 10**-**fold with minimal variation.

### The EZMTT reagent in the assay exhibits no toxicity to Gram-negative bacteria and enhances detection sensitivity by 5- to 20-fold

**Fig 3:**
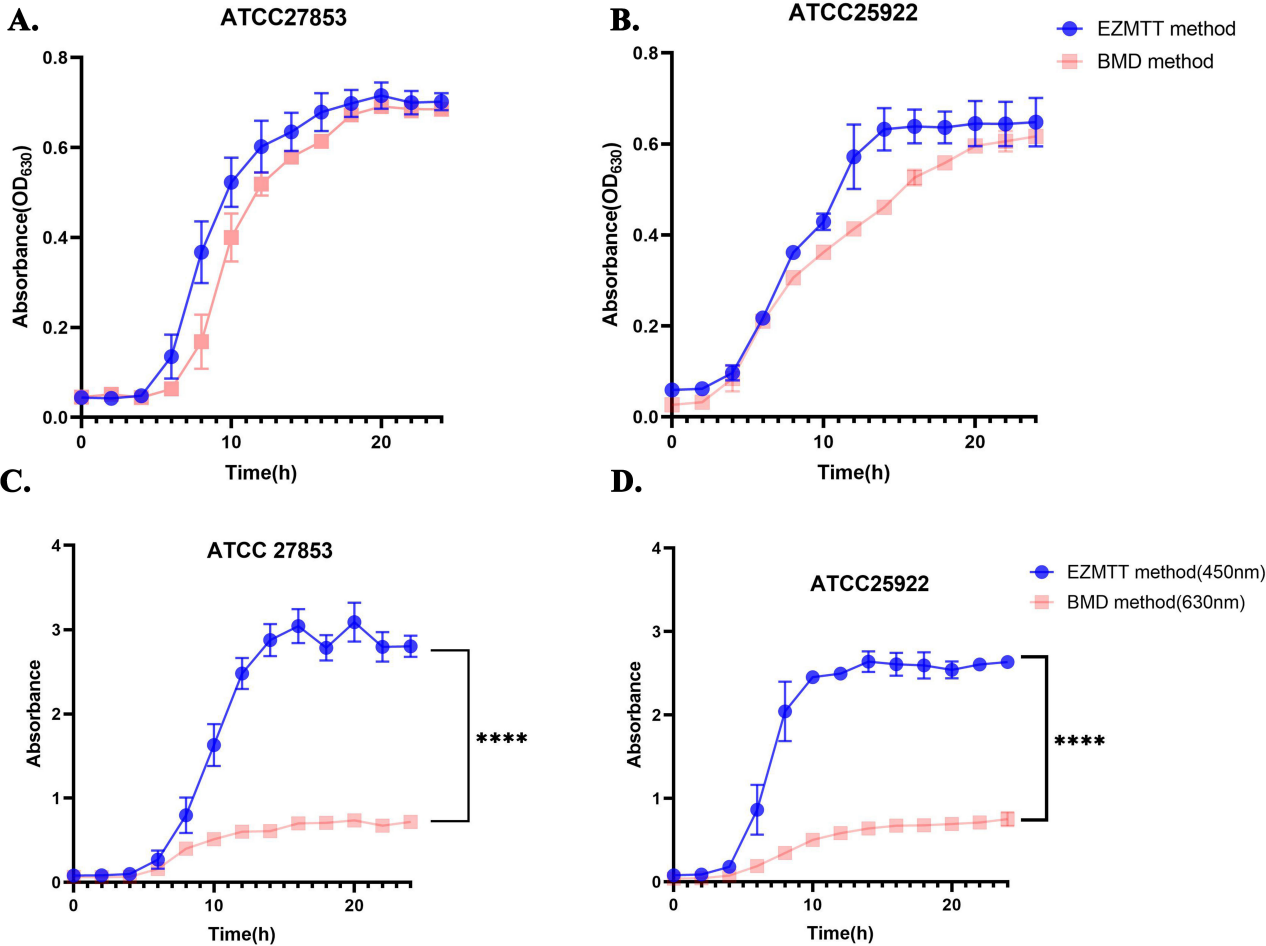
Growth curves of the QC strains (*E. coli*. ATCC25922 and *P. aerogen* ATCC27853) by the BMD method in the presence or absence of the EZMTT. (A and B) The growth of both bacterial strains was measured at 630 nm, and essentially the same dose‒response curves were observed in the presence or absence of EZMTT, with a *P* value of > 0.05. (C and D) The growth of both bacterial strains was measured at 450 nm or 630 nm, in the presence or absence of the EZMTT, respectively. Statistical analysis revealed a dramatic signal increase in the EZMTT method, with a *P* value of < 0.0001 (****).

The bacterial growth curves were measured by the bacteria cell turbidity (OD_630_nm) using broth microdilution (BMD) in the presence or absence of the EZMTT indicator, as illustrated in Fig. 3A and B; the growth curves measured at 630 nm for the quality control (QC) strains (ATCC25922 or ATCC27853) with or without the EZMTT indicator were virtually identical, suggesting that EZMTT does not exhibit toxicity toward the tested strains. Under the same conditions, EZMTT also showed non-toxic effects toward several other strains, including *Enterobacter cloacae, K. pneumoniae, Citrobacter* spp*., Proteus* spp*, A. baumannii,* and *S. maltophilia* (Fig. S2). When the growth signals were measured at the optimal growth signal for the EZMTT (OD_450nm_, orange–red color) or the turbidity at OD_630nm_ (cell density), Fig. 3C and D; Fig. S3 demonstrate that the addition of EZMTT resulted in a 5- to 20-fold enhancement of the growth signal across 10 different Gram-negative bacterial strains tested.

### The precision experiment demonstrated favorable intra- and inter-batch consistency for the EZMTT method

**Fig 4:**
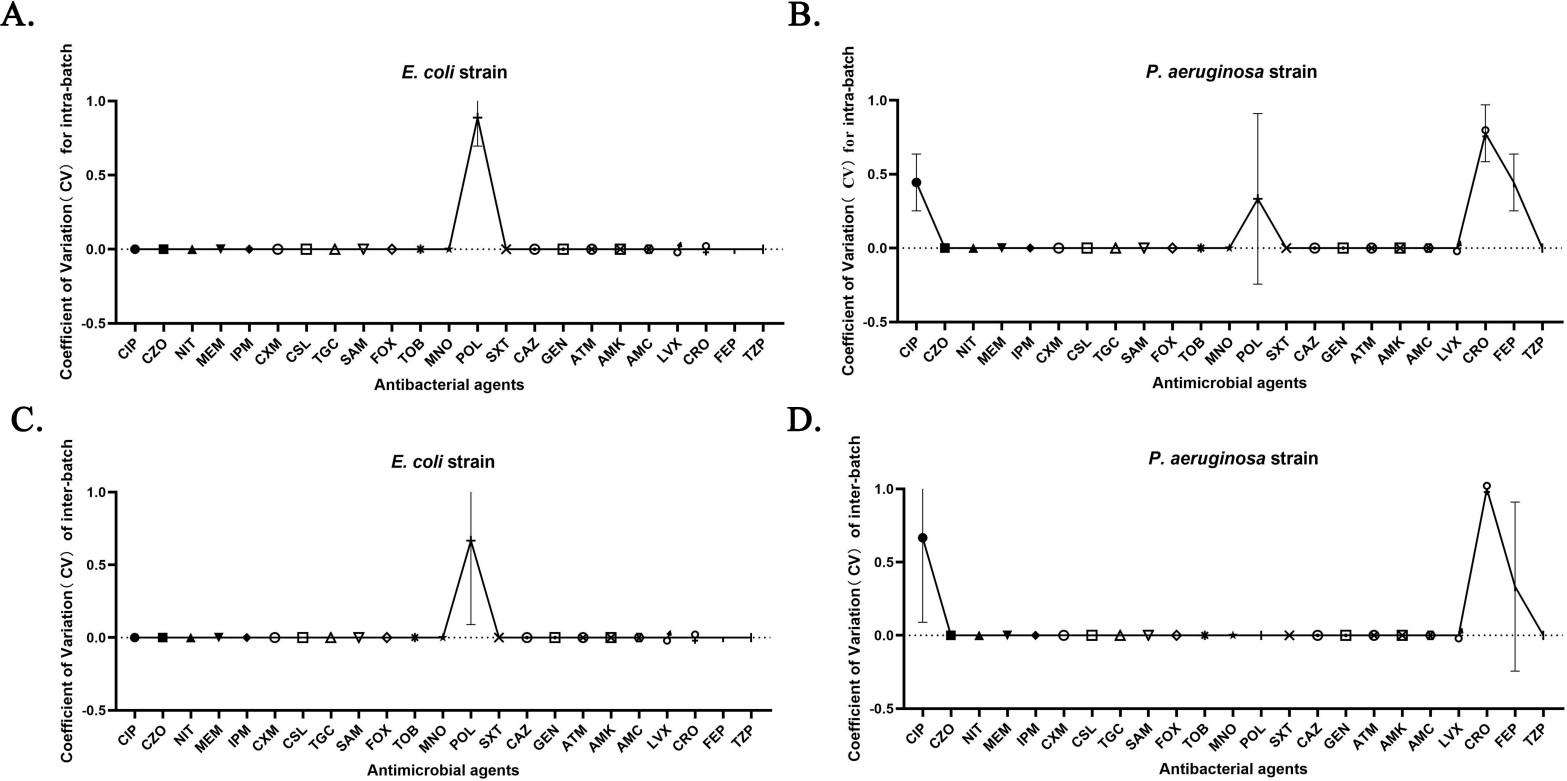
Precision and reproducibility in MIC measurement by the EZMTT method. (**A**) intra-batch difference in the *E. coli* strain and (**B**) intra-batch difference in the *P aeruginosa* strain. (**C**) interbatch difference in *E. coli* (**D**) interbatch difference in *P. aeruginosa*. CIP: Ciprofloxacin; CZO: Cefazolin; NIT: Nitrofurantoin; MEM: Meropenem; IPM: Imipenem; CXM: Cefuroxime; CSL: Cefoperazone sulbactam; TGC: Tigecycline; SAM: Ampicillin/sulbactam; FOX: Cefoxitin; TOB: Tobramycin; MNO: Minocycline; POL: Polymyxin; SXT: Trimethoprim/sulfamethoxazole; CAZ: Ceftazidime; GEN: Gentamicin; ATM: Aztreonam; AMK: Amikacin; AMC: Amoxicillin-clavulanic acid; LVX: Levofloxacin; CRO: Ceftriaxone; FEP: Cefepime; TZP: Piperacillin/Tazobactam.

The results presented in Fig. 3 indicate that the EZMTT assay is a potentially effective method for rapid detection of bacterial growth, although its assay reproducibility requires validation. Precision and reproducibility tests were performed using three batches of assay plates (GN#1, GN#2, and GN#3; GN) containing 23 antibiotics and the two QC strains (*E. coli* and *P. aeruginosa)*. As demonstrated in Table S1, the MIC values obtained by the EZMTT method align with the CLSI predetermined MIC values from the BMD assay for both QC strains (21). Furthermore, Figure 4 illustrates that the precision of the EZMTT method in 276 tests meets the evaluation criteria; the coefficients of variation (CVs) for most antibacterial drugs are zero, indicating exceptionally high reproducibility. Other CVs within 1% are observed for CIP, POL, CRO, and FEP, but their observed intra-batch difference remains within one dilution concentration variation and inter-batches within ±1 dilution. Overall, both intra-batch and inter-batch difference tests for *E. coli* yielded identical MIC values for 22 antibiotics, except for POL (1% CV), while tests for *P. aeruginosa* showed consistency for 19 antibiotics, except for CIP, POL, CRO, and FEP (1% CV).

### Validation of the EZMTT method

The BMD method is considered the gold standard for AST. To validate the consistency of the BMD method in the presence or absence of the EZMTT, we performed a comparative analysis using clinical bacterial strains. As presented in Table 2, among the 207 clinical strains examined, 153 strains demonstrated 100% consistency, 43 strains showed 95% consistency, and 11 strains exhibited less than 95% consistency between the two methods. The calculated 95% identity agreement rate is 94.7%, with only 5.3% of strains presenting multiple divergent AST results.

**TABLE 2:** Comparison of the AST consistency between the EZMTT and the traditional BMD methods (*n* = 207)

Method types	Total sample number (n)	Consistency percentage					
		100%		95%		<95%	
		Number (n)	Proportion	Number (n)	Proportion	Number (n)	Proportion
EZMTT	207	153	73.2%	43	20.8%	11	5.3%
BMD							

Consistency analyses were conducted at each drug level for every bacterial strain. Using the weighted average method, the positive compliance rate between both methods was 98.94%, the negative compliance rate was 99.58%, and the kappa value was 0.981, with a 95% CI of 0.974–0.989, indicating strong classification consistency. According to the FDA criteria (22), the current clinical comparison results demonstrated consistent AST results that met the general authoritative standards. For 22 of the 23 antibiotics tested, the compliance rate exceeded 90.00%, with MNO being the exception. The kappa values for 21 compounds ranged from 0.794 to 1.000, while CXM and TGC had lower kappa values of 0.479 and 0.489, respectively (Table 3). These results suggest that the BMD method, both with and without the EZMTT indicator, demonstrates good consistency and adheres to CLSI breakpoint standards.

**TABLE 3:** Statistical analysis of AST results collected for 23 antibiotics by the EZMTT-based and the control BMD methods

	Number (n)	Positive conformity rate (%)	Negative conformity rate (%)	Positive predictive value (%)	Negative predictive value (%)	Total consistent rate (%)	kappa value	*Z* value	*P* value
CIP	177	100.0	100.0	100.0	100.0	100.0	1	63.25	<0.05
CZO	92	100.0	93.75	97.10	100.0	97.98	0.953	29.04	<0.05
NIT	100	95.56	98.04	97.73	96.15	96.88	0.937	26.26	<0.05
MEM	195	99.10	100.0	100.0	98.84	99.49	0.990	95.66	<0.05
IPM	192	91.13	100.0	100.0	98.73	99.48	0.989	93.09	<0.05
CXM	73	100.0	33.33	94.37	100.0	94.52	0.479	2.21	<0.05
CSL	96	97.96	98.00	97.96	98.00	97.98	0.960	33.93	<0.05
TGC	95	33.33	100.0	100.0	97.20	97.24	0.489	3.04	<0.05
SAM	140	100.0	100.0	100.0	100.0	100.0	1	45.61	<0.05
FOX	94	96.00	95.74	96.00	95.74	95.88	0.917	22.70	<0.05
TOB	199	100.0	100.0	100.0	100.0	100.0	1	41.32	<0.05
MNO	113	85.94	95.45	96.49	82.35	89.81	0.794	12.65	<0.05
POL	205	100.0	99.48	94.12	100.0	99.52	0.967	29.46	<0.05
SXT	158	94.23	96.23	98.00	89.47	94.90	0.888	23.1	<0.05
CAZ	188	77.78	100.0	100.0	90.41	92.82	0.826	18.69	<0.05
GEN	189	98.85	100.0	100.0	99.04	99.47	0.989	93.49	<0.05
ATM	165	100.0	100.0	100.0	100.0	100.0	1	54.27	<0.05
AMK	189	100.0	100.0	100.0	100.0	100.0	1	48.76	<0.05
AMC	101	90.00	97.92	97.83	90.38	93.88	0.878	18.2	<0.05
LVX	177	97.60	98.28	99.19	95.00	97.81	0.950	38.42	<0.05
CRO	149	100.0	100.0	100.0	100.0	100.0	1	42.54	<0.05
FEP	194	99.17	100.0	100.0	98.70	99.49	0.989	92.83	<0.05
TZP	148	100.0	100.0	100.0	100.0	100.0	1	24.72	<0.05

The VITEK and K-B methods are the most widely used clinical approaches for AST. Consequently, ASTs were conducted using VITEK, K-B, broth microdilution (BMD), and EZMTT methods for the clinical bacterial strains EC203, EC211, EC227, EC238, KP219, and KP260. The findings revealed that the EZMTT method’s AST results were entirely consistent with those of the other methods for all bacterial strains except KP260. In the case of KP260, where a differential result (I versus S) was observed for TZP, the EZMTT method maintained consistency with one of the other methods (Table 4).

**TABLE 4:** Comparison of AST results for commonly used clinical methods (VITEK or K-B methods), the BMD method, and the EZMTT method in clinical *E. coli* (EC), and *K. pneumoniae* strains (KP) (*n* = 6)

Antimicrobial agents	EC203			EC211			EC227			EC238			KP219			KP260		
	EZ^*a*^	TR^*b*^	CL^*c*^	EZ^*a*^	TR^*b*^	CL^*c*^	EZ^*a*^	TR^*b*^	CL^*c*^	EZ^*a*^	TR^*b*^	CL^*c*^	EZ^*a*^	TR^*b*^	CL^*c*^	EZ^*a*^	TR^*b*^	CL^*c*^
TZP	S	S	S^*d*^	S	S	S^*d*^	R	R	R^*d*^	S	S	S^*d*^	S	S	S^*d*^	**I**	**I**	**S** ^*d*^
CZO	S	S	S^*d*^	S	S	S^*d*^	R	R	R^*d*^	S	S	S^*d*^	S	S	S^*d*^	S	S	S^*d*^
CAZ	S	S	S^*d*^	S	S	S^*d*^	R	R	R^*d*^	S	S	S^*d*^	S	S	S^*d*^	S	S	S^*d*^
CRO	S	S	S^*d*^	S	S	S^*d*^	R	R	R^*d*^	S	S	S^*d*^	S	S	S^*d*^	S	S	S^*d*^
FEP	S	S	S^*d*^	S	S	S^*d*^	R	R	R^*d*^	S	S	S^*d*^	S	S	S^*d*^	S	S	S^*d*^
ATM	S	S	S^*d*^	S	S	S^*d*^	S	S	S^*d*^	S	S	S^*d*^	S	S	S^*d*^	S	S	S^*d*^
MEM	S	S	S^*e*^	S	S	S^*e*^	R	R	R^*e*^	S	S	S^*e*^	S	S	S^*e*^	S	S	S^*e*^
IPM	S	S	S^*e*^	S	S	S^*e*^	R	R	R^*e*^	S	S	S^*e*^	S	S	S^*e*^	**S**	**I**	**S** ^*e*^
GEN	R	R	R^*d*^	S	S	S^*d*^	R	R	R^*d*^	S	S	S^*d*^	S	S	S^*d*^	S	S	S^*d*^
TOB	**R**	**R**	**I** ^*d*^	S	S	S^*d*^	I	I	I^*d*^	S	S	S^*d*^	S	S	S^*d*^	S	S	S^*d*^
AMK	S	S	S^*d*^	S	S	S^*d*^	S	S	S^*d*^	S	S	S^*d*^	S	S	S^*d*^	S	S	S^*d*^

^*a*^ EZMTT method.

^*b*^ BMD method.

^*c*^ Commonly used clinical methods.

^*d*^ VITEK method (COMPACT automated analyzer).

^*e*^ K-B method. S: sensitive; R: resistant; I: intermediary. The bold and underlined values indicate inconsistent AST results among the EZ, TR, and CL methods.

### Isolation of drug-resistant bacterial subpopulations and DNA sequencing of clinical bacteria

Resistant subpopulations were isolated from the 11 clinical strains exhibiting less than 95% consistency (Table 2), and their corresponding homologous resistant strains were isolated from the inhibition zone of the K-B method and validated via mass spectrometry (VITEC-MS). Among the four bacterial strains (EC001, EZ003, KP007, and PA004) identified, KP007 demonstrated heteroresistance to MEM, detectable by the EZMTT method but not by the BMD method. The current single detection method is suboptimal for carbapenem resistance detection (23). KP007 was selected to isolate its drug-resistant subpopulation and primary drug-sensitive population on CAM-MH solid media plates with or without MEM. Consequently, the corresponding sensitive strain (KP007-S_MEM_) and resistant strain (KP007-R_MEM_) were isolated for DNA sequencing to validate the consistency between genotyping and phenotyping assays. The whole genomes of the isolated multidrug-resistant KP007 (KP007-R) and multidrug-sensitive KP007 (KP007-S) strains were analyzed via the CARD GenBank. The analysis revealed that the KP007-R and KP007-S genomes carried 39 and 28 drug resistance genes, respectively, including genes that can develop drug resistance through four mechanisms: alterations in drug targets, drug efflux pumps, drug inactivation, and reduction in drug permeability. The AST results (Table 6) indicated that strain KP007-R was resistant to various antimicrobial drugs, including β-lactams, carbapenems, quinolones, and sulfonamides. A comparison of specific drug resistance genes carried by both bacterial strains revealed that they shared most resistance genes, except for *NDM-1*, *QnrS1*, *Sul1*, *dfrA27*, *AAC(3)-Ib/AAC(6')-Ib''*, *catB3*, *BRP(MBL)*, *aadA16*, *arr-3* and *AAC(6')-Ib10* genes, which were present in KP007-R but absent in KP007-S (Table 5). These findings suggest that the bacterial strains and their drug-resistant bacterial subpopulations indeed exhibit significant differences according to the EZMTT method, thus providing a foundation for future studies on the application of this method for detecting drug-resistant bacterial subpopulations.

**TABLE 5:** Comparison of the genotype and phenotype consistency of the clinical KP007-R and KP007-S bacterial strains

Antimicrobial agents	EZMTT		DNA sequencing			
	KP007-R	KP007-S	Drug resistance genes	Drug-resistant mechanisms	Bacterial strains	
					KP007-R	KP007-S
Beta-lactams			*QnrS1*	Antibiotic target protection	YES	NO
Cefazolin	R	S	*AAC(3)-Ib/AAC(6')-Ib''*	Antibiotic inactivation	YES	NO
Cefuroxime	R	S	*sul1*	Antibiotic target replacement	YES	NO
Cefoperazone/sulbactam	R	S	*catB3*	Antibiotic inactivation	YES	NO
Cefoxitin	R	S	*NDM-1*	Antibiotic inactivation	YES	NO
Ceftazidime	R	S	*BRP(MBL)*	Antibiotic inactivation	YES	NO
Ceftriaxone	R	S	*sul1*	Antibiotic target replacement	YES	NO
Cefepime	R	S	*aadA16*	Antibiotic inactivation	YES	NO
Piperacillin/tazobactam	R	I	*dfrA27*	Antibiotic target replacement	YES	NO
Carbapenems			*arr-3*	Antibiotic inactivation	YES	NO
Meropenem	R	S	*AAC(6')-Ib10*	Antibiotic inactivation	YES	NO
Imipenem	R	S	*catB3*	Antibiotic inactivation	YES	NO
Quinolones			*NDM-1*	Antibiotic inactivation	YES	NO
Ciprofloxacin	R	S				
Sulfonamides						
Cotrimoxazole	R	S				

### The EZMTT method demonstrates efficacy in detecting heteroresistant bacterial colonies in clinical settings

Given that the KP007-S_MEM_ and KP007-R_MEM_ strains identified through DNA sequencing are relatively pure strains, while the parent KP007 contains drug-resistant subpopulations, we conducted AST in comparison with commonly used clinical AST methods. As illustrated in Table 6, the relatively pure strains KP007-S_MEM_ and KP007-R_MEM_ demonstrated essentially identical AST results for 18 antibiotics (95% agreement) between the EZMTT and broth microdilution (BMD) methods, with the exception of MNO and piperacillin-tazobactam (TZP), for which the EZMTT method indicated “Intermediate,” whereas the BMD method showed “Susceptible.” The K-B method exhibited only 85% agreement with the EZMTT and BMD methods, with discrepancies observed for NIT, TOB, MNO, cefazolin (CZO), piperacillin-tazobactam (TZP), and POL.

**TABLE 6:** The AST results of the KP007, KP007-R_MEM_, and KP007-S_MEM_ isolates were compared via the K-B, EZMTT, and BMD methods

Antimicrobial agents	K-B method of folding points (mm）		KP007				KP007-R_MEM_			KP007-S_MEM_		
	S	R	K-B^*a*^	EZ^*b*^	TR^*c*^	CL^*d*^	K-B^*a*^	EZ^*b*^	TR^*c*^	K-B^*a*^	EZ^*b*^	TR^*c*^
CIP	≥26	≤ 21	**HR**	**R**	**I**	**I^*e*^**	R	R	R	S	S	S
CZO	≥23	≤ 19	**HR**	**R**	**R**	**R^*e*^**	R	R	R	**I**	S	S
NIT	≥17	≤ 14	R	R	R	I^*e*^	**I**	R	R	S	S	S
MEM	≥23	≤ 19	**HR**	**R**	**S**	**R^*a*^**	**HR**	R	R	S	S	S
IPM	≥23	≤ 19	R	R	R	R**^*a*^**	R	R	R	S	S	S
CXM	≥23	≤ 14	**HR**	**R**	**R**	-	R	R	R	S	S	S
CSL	≥21	≤ 15	**HR**	**R**	**R**	**R^*a*^**	R	R	R	S	S	S
FOX	≥18	≤ 14	**HR**	**R**	**R**	-	R	R	R	S	S	S
TOB	≥15	≤ 12	**HR**	**R**	**R**	**I^*e*^**	**S**	I	I	S	S	S
MNO	≥16	≤ 12	**R**	**R**	**I**	-	**R**	I	*S*	**I**	S	S
POL	-		-	R	R	-	-	I	I	-	I	I
SXT	≥16	≤ 10	**HR**	**R**	**R**	**R^*e*^**	R	R	R	S	S	S
CAZ	≥21	≤ 17	R	R	R	R**^*a*^**	R	R	R	S	S	S
GEN	≥15	≤ 12	**HR**	**R**	**R**	**S^*e*^**	S	S	S	S	S	S
ATM	≥21	≤ 17	**HR**	**R**	**R**	**S^*e*^**	S	S	S	S	S	S
AMK	≥17	≤ 14	**HR**	**R**	**S**	**S^*e*^**	S	S	S	S	S	S
LVX	≥21	≤ 16	S	S	S	I^*e*^	S	S	S	S	S	S
CRO	≥23	≤ 19	R	R	R	R**^*e*^**	R	R	R	S	S	S
FEP	≥25	≤ 18	**HR**	**R**	**R**	**R^*e*^**	R	R	R	S	S	S
TZP	≥21	≤ 17	**HR**	**R**	**R**	**R^*e*^**	R	R	R	S	**I**	*S*

^*a*^ K-B method.

^*b*^ EZMTT method.

^*c*^ BMD method.

^*d*^ Commonly used clinical methods.

^*e*^ VITEK method (COMPACT automated analyzer). S: sensitive; R: resistant; I: intermediary; HR: hidden resistant. “-” not applicable. The bold values indicate inconsistent AST results among the K-B，EZ, TR, and CL methods.

The most interesting data were obtained from the parent KP007 strain, which contains drug-resistant subpopulations for 13 antibiotics as measured by the K-B method, as shown in Table 6. The EZMTT method identified all 13 heteroresistance cases as “R resistant” (representing a 100% hit rate), with other R and S results agreeing with the K-B method. The broth microdilution (BMD) method identified 10 heteroresistance cases as “R resistant” (a 77% hit rate), 1 as “I intermediate,” and 2 as “S sensitive.” Unexpectedly, the VITEK AST method only identified four heteroresistance cases as “R resistant,” two as “I intermediate, three as "S sensitive”, and four as not available. Considering that VITEK AST is the predominant AST method used in the majority of hospitals worldwide, this less than 44% (4 out of 9) hit rate is notably low. Further investigation is warranted to determine the sensitivity of the VITEK AST method in detecting drug-resistant subpopulations.

**Fig 5:**
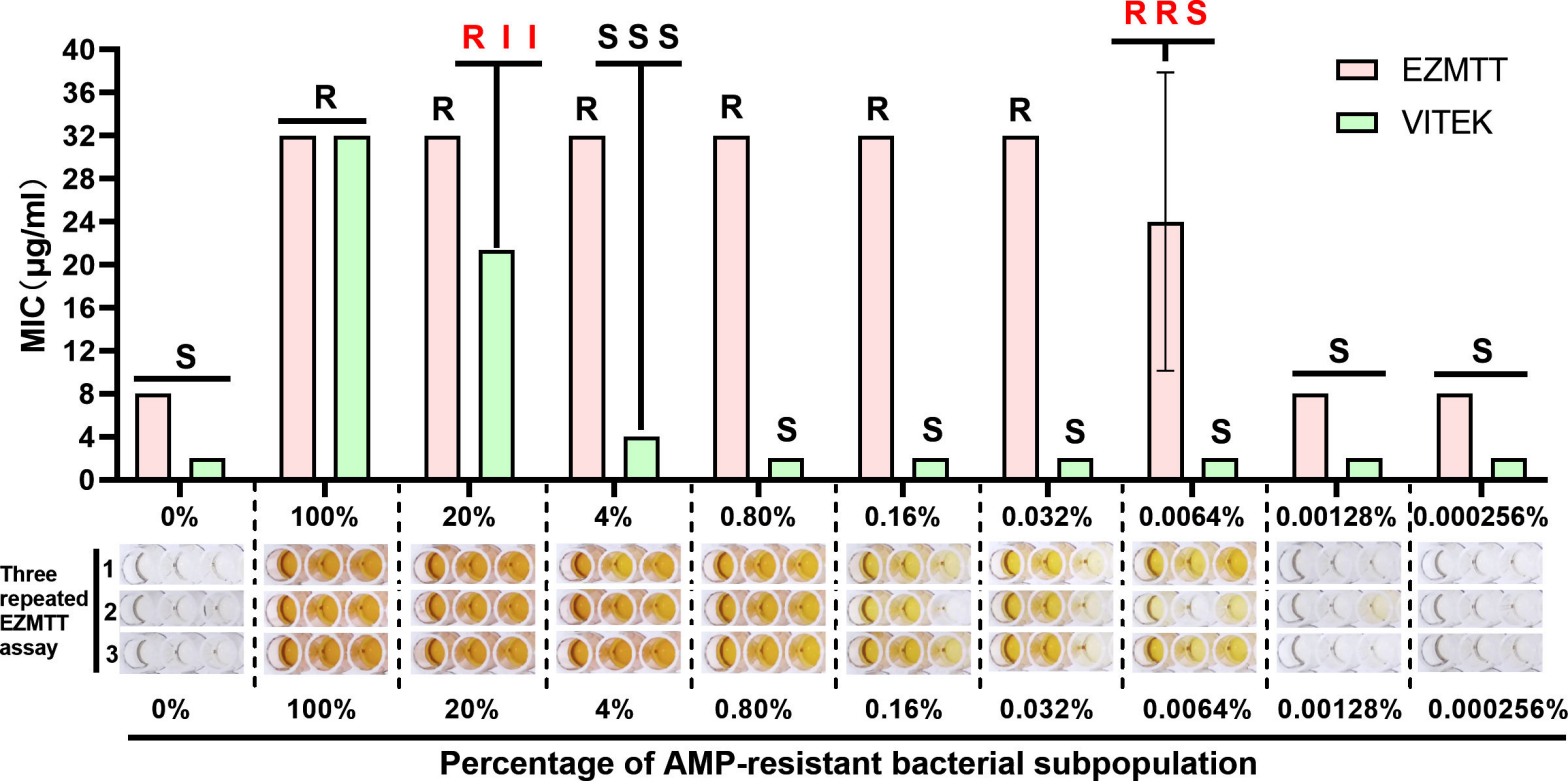
Comparison of the EZMTT and VITEK methods for detecting drug-resistant bacterial subpopulations. Note: R: resistance, I: mediation, S: sensitivity, AMP: ampicillin; VITEK: VITEK 2 Compact Drug Sensitivity Analyzer (Merieux, France).

A pair of ampicillin-resistant and ampicillin-sensitive *E. coli* strains was created by transducing the *E. coli* DH10B strain with an ampicillin resistance gene (DH10B and DH10B-Amp^R^). To compare the sensitivity of the VITEK and EZMTT AST methods in detecting the ampicillin-resistant subpopulation, the sensitive strain (DH10B) was freshly mixed with a series of fivefold dilutions of the resistant strain (DH10B-Amp^R^). The mixed strains were immediately subjected to AST using the VITEK (AST-GN13) and EZMTT-based methods according to the manufacturer’s instructions. As illustrated in Fig. 5, the VITEK 2 (AST-GN13) method detected the 20% resistant bacterial subpopulation with slight variation (R, I, I), meeting the expected detection limit for a BMD method. By contrast, the EZMTT-based method provided unambiguous detection (“R, resistant” in all three repeated tests) of resistant bacterial subpopulations ranging from 20% to 0.032%. The detection limit for the EZMTT method was 0.0064% (R, S, R), with the absence of growth in the middle well potentially resulting from an insufficient number of resistant bacteria being dispensed in each well of a 96-well plate. In summary, the EZMTT-based AST method for Gram-negative bacteria demonstrated over 3,000 times greater effectiveness in detecting the resistant bacterial subpopulation compared to the VITEK method (Fig. 5).

## DISCUSSION

Over the past three decades, instrumental high-throughput AST methods have been developed and widely implemented in clinical settings. Nevertheless, bacterial resistance remains a significant medical and societal concern globally. According to the Chinese Antimicrobial Resistance Surveillance System in 2020, the average resistance rate of bacteria to certain antimicrobial agents currently ranges from 18% to 53%. In response to the increasing prevalence of antibiotic-resistant bacteria, antibacterial agents with novel mechanisms of action have been developed (24–26). Unfortunately, resistant bacteria typically emerge shortly after a new antibiotic enters the market. Consequently, the prevention and control of drug-resistant bacteria have become increasingly critical and are now recognized as global issues. The rational use of antimicrobial drugs is urgently needed to decelerate the emergence of antimicrobial resistance (AMR) in bacteria. Furthermore, there is an intense demand in clinical settings for AST methods with improved sensitivity and accuracy.

Cell viability and proliferation assessment are crucial components of cytological research. Our research team developed EZMTT, a monosulfate tetrazolium salt (18). This compound offers advantages over the MTT method due to its non-cytotoxic, water-soluble, and stable natures (5, 27), and the soluble orange-red colored formazan produced by its reaction with the cofactor NAD(P)H of living cells showed a strong signal-to-background ratio and significantly enhanced detection signals. The detection method exhibits a linear dose response to NAD(P)H, dehydrogenase concentration, and cell number. Unlike the MTS, XTT, and WST methods, false positives do not occur in the presence of antioxidants such as BME or GSH. Consequently, this detection method provides a broader detection range, improved MIC reproducibility, and reduced antioxidant side reactions. In addition, it offers the benefits of cost-effectiveness, enhanced accuracy, and precision (19).

The BMD method is currently considered reliable for determining bacterial drug sensitivity (28) and serves as the reference method for CLSI breakpoint standards. We conducted various AST comparisons using the BMD method, both with and without the EZMTT indicator. The quality control strains *E. cloacae* ATCC25922 and *P. aerogen* ATCC27853 demonstrated good accuracy and precision in MIC values, comparable to the BMD values listed in CLSI-M100S. In addition, testing using 207 clinical strains showed consistent MIC results between the BMD and EZMTT method. Thiswhich suggests that the breakpoints from CLSI-M100S are appropriate for the EZMTT method (29). Moreover, when employing the CLSI-M100S breakpoints, strains without resistant subpopulations exhibited essentially identical AST(R/S) results across the EZMTT, BMD, and VITEK methods (18, 29, 30). Collectively, these findings validate the use of CLSI-M100S breakpoints for AST in the EZMTT-based method.

The EZMTT method’s significant advantage lies in its ability to amplify the living bacteria signal by 5–20 times, enabling more sensitive detection and precise AST results in combination with automation, thus identifying trace-level “hidden” heterogeneous drug-resistant bacteria in high-throughput screening by BMD assay in the presence or absence of the EZMTT agent (e.g., Table 2; <95% group). For instance, the EZMTT results of cefuroxime testing revealed that >90% of strains were resistant, with the remaining strains containing a resistant subpopulation undetected by the BMD method. Moreover, while K-B methods identified 13 drug-resistant subpopulations in the clinical *K. pneumoniae* KP007 strain, the EZMTT method demonstrated 100% detection, compared to 77% for BMD and less than 44% for VITEK. This detection rate for resistant subpopulations aligns with the observed 3,000-fold superior sensitivity in AMP^R^ subpopulation detection by EZMTT compared to VITEK methods.

The severity of drug resistance is influenced by various factors, including the misuse of antibacterial agents and excessive treatment (31). While the mechanism of bacterial drug resistance is complex, the emergence of bacterial heteroresistance is a significant contributing factor (32). Bacterial heteroresistance typically refers to the presence of subpopulations within a single isolated strain that exhibit varying sensitivities to a particular drug in its cultured population. In such cases, some cells demonstrate sensitivity to the drug, while others display resistance, leading to the classification of these bacteria as heterogeneously resistant strains. This heterogeneity in bacterial resistance presents substantial challenges in evaluating the therapeutic efficacy of drugs against pathogenic bacteria (33, 34) and diminishes the reliability of laboratory MIC data (35). Consequently, these heteroresistant bacteria may result in treatment failures, exacerbate infections, and potentially pose greater public health risks during treatment (36). There is an urgent need for improved sensitive detection methods to identify resistant bacterial subpopulations. We hypothesized that the limited sensitivity of traditional clinical AST in detecting resistant subpopulations may contribute to the transformation of these subpopulations into multidrug-resistant strains. Therefore, the application of the EZMTT method to identify the resistant subpopulations in gram-negative bacteria could potentially provide clinicians with a more comprehensive and precise AST, although its future application in precision medicine needs to be established. In addition, the application of the EZMTT method to Gram-negative bacteria grown in CAM-MH culture media has been tested, but bacteria species with special requirements remain to be investigated in the future.

In conclusion, the EZMTT method we developed demonstrated superior accuracy, precision, and sensitivity in Gram-negative bacteria AST; as long as a strain does not contain minor levels of resistant subpopulation, the EZMTT, BMD, and VITEK methods showed good consistency. Based on AST results of 207 clinical Gram-negative bacteria tested, approximately 5%–10% of bacteria might exist as small numbers of resistant subpopulations. As a highly sensitive method, the EZMTT method exhibited 3,000 times greater sensitivity in detecting ampicillin-resistant subpopulations in *E. coli* than the VITEK method and achieved 100% detection of various heteroresistant subpopulations identified by the K-B method. Furthermore, the EZMTT method incorporates 23 commonly used antimicrobial drugs in a 96-well plate, offering a wider range of antibiotic options, as well as low-cost and high-throughput automation in reagent addition and data processing, enablinging efficiency and accuracy in testing and improved cost-effectiveness. In addition, our early investigation showed that the EZMTT reagent is applicable to a wide range of pathogens, including Gram-negative bacteria, Gram-positive bacteria, fungi, and *Mycoplasma bacteria.* Taken together, the EZMTT-based AST method shows promise as a valuable tool for managing multidrug-resistant bacteria and has potential applications in precision medicine.

## Data Availability

All data utilized in this research are accessible upon request. The draft genomes of the K. pneumoniae isolates sequenced in our study have been deposited in the NCBI database under BioProject PRJNA1259721 (KP007-S: GCA_050376235.1, KP007-R: GCA_050376215.1).

## SUPPLEMENTAL MATERIAL

Supplemental figures spectrum.00011-25-s0001.docx Figures S1 to S3.

Table S1 spectrum.00011-25-s0002.docx Comparison of AST results from both types of assay plates by the EZMTT assay with CLSI standards.

ASM does not own the copyrights to Supplemental Material that may be linked to, or accessed through, an article. The authors have granted ASM a non-exclusive, world-wide license to publish the Supplemental Material files. Please contact the corresponding author directly for reuse.

## References

[1] Arzanlou M, Chai WC, Venter H. 2017. Intrinsic, adaptive and acquired antimicrobial resistance in Gram-negative bacteria. Essays Biochem 61:49–59. doi:10.1042/EBC20160063 28258229

[2] Xuan J, Feng W, Wang J, Wang R, Zhang B, Bo L, Chen Z-S, Yang H, Sun L. 2023. Antimicrobial peptides for combating drug-resistant bacterial infections. Drug Resist Updat 68:100954. doi:10.1016/j.drup.2023.100954 36905712

[3] Smith KP, Kirby JE. 2019. Rapid susceptibility testing methods. Clin Lab Med 39:333–344. doi:10.1016/j.cll.2019.04.001 31383260 PMC6686868

[4] Balouiri M, Sadiki M, Ibnsouda SK. 2016. Methods for in vitro evaluating antimicrobial activity: a review. J Pharm Anal 6:71–79. doi:10.1016/j.jpha.2015.11.005 29403965 PMC5762448

[5] Chernogor L, Bakhvalova K, Belikova A, Belikov S. 2022. Isolation and properties of the bacterial strain Janthinobacterium sp. SLB01. Microorganisms 10:1071. doi:10.3390/microorganisms10051071 35630513 PMC9147652

[6] Demir C, Keşli R. 2018. Identification of anaerobic Gram-negative bacilli isolated from various clinical specimens and determination of antibiotic resistance profiles with E-test methods. Mikrobiyol Bul 52:72–79. doi:10.5578/mb.66175 29642831

[7] Lo TS, Goto M, Hammer KDP. 2024. Evaluating the performance of the Alere PBP2a SA culture colony test with the Vitek 2 antimicrobial susceptibility test card system as reference standard in coagulase-negative Staphylococcus species. Infect Med (Beijing) 3:100126. doi:10.1016/j.imj.2024.100126 39291080 PMC11405896

[8] Krüger A, Körber-Irrgang B, Flüh G, Gielen J, Scholz C-J, Wisplinghoff H, Jazmati N. 2024. Rapid antimicrobial susceptibility testing using the MicroScan System: performance evaluation of a 4-hour bacterial cultivation from positive blood cultures. Curr Microbiol 81:261. doi:10.1007/s00284-024-03768-9 38981918

[9] Sieswerda E, Bosch T, Lankelma JM, Schouls LM, van Dijk K. 2021. Vitek 2 MICs as first-line phenotypic screening method for carbapenemase-producing Pseudomonas aeruginosa. Future Microbiol 16:777–781. doi:10.2217/fmb-2020-0024 34229445

[10] Mal PB, Jabeen K, Farooqi J, Unemo M, Khan E. 2016. Antimicrobial susceptibility testing of Neisseria gonorrhoeae isolates in Pakistan by Etest compared to calibrated dichotomous sensitivity and clinical laboratory standards institute disc diffusion techniques. BMC Microbiol 16:236. doi:10.1186/s12866-016-0707-6 27724873 PMC5057452

[11] Nicoloff H, Hjort K, Levin BR, Andersson DI. 2019. The high prevalence of antibiotic heteroresistance in pathogenic bacteria is mainly caused by gene amplification. Nat Microbiol 4:504–514. doi:10.1038/s41564-018-0342-0 30742072

[12] Ernst CM, Braxton JR, Rodriguez-Osorio CA, Zagieboylo AP, Li L, Pironti A, Manson AL, Nair AV, Benson M, Cummins K, Clatworthy AE, Earl AM, Cosimi LA, Hung DT. 2020. Adaptive evolution of virulence and persistence in carbapenem-resistant Klebsiella pneumoniae. Nat Med 26:705–711. doi:10.1038/s41591-020-0825-4 32284589 PMC9194776

[13] Moosavian M, Shoja S, Nashibi R, Ebrahimi N, Tabatabaiefar MA, Rostami S, Peymani A. 2014. Post neurosurgical meningitis due to colistin heteroresistant Acinetobacter baumannii. Jundishapur J Microbiol 7:e12287. doi:10.5812/jjm.12287 25632326 PMC4295316

[14] Tan M, Yi X, Liao C, Zhou Z, Ren B, Liang L, Li X, Wei G. 2024. Establishment of a platform based on dual RPA combined with CRISPR/Cas12a for the detection of Klebsiella pneumoniae and its KPC resistance gene. Front Bioeng Biotechnol 12:1447963. doi:10.3389/fbioe.2024.1447963 39416281 PMC11480703

[15] Kontopidou F, Galani I, Panagea T, Antoniadou A, Souli M, Paramythiotou E, Koukos G, Karadani I, Armaganidis A, Giamarellou H. 2011. Comparison of direct antimicrobial susceptibility testing methods for rapid analysis of bronchial secretion samples in ventilator-associated pneumonia. Int J Antimicrob Agents 38:130–134. doi:10.1016/j.ijantimicag.2011.04.011 21658915

[16] Boyer A, Medrano J, Mzali F, Balick-Weber C-C, Bessède E, Picard W, Clouzeau B, Bébéar CM, Vargas F, Hilbert G, Rogues AM, Gruson D. 2012. Direct testing of bronchoalveolar lavages from ventilator-associated pneumonia patients. Diagn Microbiol Infect Dis 73:107–110. doi:10.1016/j.diagmicrobio.2012.02.017 22483191

[17] Lü L, Zhang L, Wai MSM, Yew DTW, Xu J. 2012. Exocytosis of MTT formazan could exacerbate cell injury. Toxicol In Vitro 26:636–644. doi:10.1016/j.tiv.2012.02.006 22401948

[18] Xie L, Chen Z, Liu W, Gu D, Yu Y, Chen X, Wu Y, Xu N, Xie J, Zhao G, Ruan BH. 2021. A sensitive EZMTT method provides microscale, quantitative and high-throughput evaluation of drug efficacy in the treatment of Mycobacterium tuberculosis infectious diseases. J Microbiol Methods 181:106136. doi:10.1016/j.mimet.2021.106136 33422524

[19] Zhang W, Zhu M, Wang F, Cao D, Ruan JJ, Su W, Ruan BH. 2016. Mono-sulfonated tetrazolium salt based NAD(P)H detection reagents suitable for dehydrogenase and real-time cell viability assays. Anal Biochem 509:33–40. doi:10.1016/j.ab.2016.06.026 27387057

[20] Hu Q, Yu Y, Gu D, Xie L, Chen X, Xu N, Ruan JJ, Dowson C, Ruan BH. 2019. Detection of “Hidden” antimicrobial drug resistance. ACS Infect Dis 5:1252–1263. doi:10.1021/acsinfecdis.9b00132 31243989

[21] Chinese Pharmacopoeia 9101 guidelines for validation of analytical methods. 2020. Available from: https://baipharm.chemlinked.com/regulatorydb/view/9

[22] Analytical procedures and methods validation for drugs and biologics. 2015. Available from: https://www.fda.gov/regulatory-information

[23] Lutgring JD, Limbago BM. 2016. The problem of carbapenemase-producing-carbapenem-resistant-Enterobacteriaceae detection. J Clin Microbiol 54:529–534. doi:10.1128/JCM.02771-15 26739152 PMC4767976

[24] Muñoz KA, Ulrich RJ, Vasan AK, Sinclair M, Wen P-C, Holmes JR, Lee HY, Hung C-C, Fields CJ, Tajkhorshid E, Lau GW, Hergenrother PJ. 2024. A Gram-negative-selective antibiotic that spares the gut microbiome. Nature 630:429–436. doi:10.1038/s41586-024-07502-0 38811738 PMC12135874

[25] Wang Z, Koirala B, Hernandez Y, Zimmerman M, Park S, Perlin DS, Brady SF. 2022. A naturally inspired antibiotic to target multidrug-resistant pathogens. Nature 601:606–611. doi:10.1038/s41586-021-04264-x 34987225 PMC10321319

[26] Breijyeh Z, Jubeh B, Karaman R. 2020. Resistance of Gram-negative bacteria to current antibacterial agents and approaches to resolve it. Molecules 25:1340. doi:10.3390/molecules25061340 32187986 PMC7144564

[27] Präbst K, Engelhardt H, Ringgeler S, Hübner H. 2017. Basic colorimetric proliferation assays: MTT, WST, and Resazurin. Methods Mol Biol 1601:1–17. doi:10.1007/978-1-4939-6960-9_1 28470513

[28] Vanegas D, Abril-Novillo A, Khachatryan A, Jerves-Andrade L, Peñaherrera E, Cuzco N, Wilches I, Calle J, León-Tamariz F. 2021. Validation of a method of broth microdilution for the determination of antibacterial activity of essential oils. BMC Res Notes 14:439. doi:10.1186/s13104-021-05838-8 34857039 PMC8638534

[29] 2025. Performance standards for antimicrobial susceptibility testing. 35th ed. CLSI M100. Clinical and Laboratory Standards Institute, James S. Lewis II, PharmD, FIDSA.

[30] Bobenchik AM, Deak E, Hindler JA, Charlton CL, Humphries RM. 2015. Performance of Vitek 2 for antimicrobial susceptibility testing of Enterobacteriaceae with Vitek 2 (2009 FDA) and 2014 CLSI breakpoints. J Clin Microbiol 53:816–823. doi:10.1128/JCM.02697-14 25540403 PMC4390649

[31] Mave V, Chandanwale A, Kagal A, Khadse S, Kadam D, Bharadwaj R, Dohe V, Robinson ML, Kinikar A, Joshi S, Raichur P, McIntire K, Kanade S, Sachs J, Valvi C, Balasubramanian U, Kulkarni V, Milstone AM, Marbaniang I, Zenilman J, Gupta A. 2017. High burden of antimicrobial resistance and mortality among adults and children with community-onset bacterial infections in India. J Infect Dis 215:1312–1320. doi:10.1093/infdis/jix114 28329303 PMC5853545

[32] Nicoloff H, Hjort K, Andersson DI, Wang H. 2024. Three concurrent mechanisms generate gene copy number variation and transient antibiotic heteroresistance. Nat Commun 15:3981. doi:10.1038/s41467-024-48233-0 38730266 PMC11087502

[33] Howard-Anderson J, Davis M, Page AM, Bower CW, Smith G, Jacob JT, Andersson DI, Weiss DS, Satola SW. 2022. Prevalence of colistin heteroresistance in carbapenem-resistant Pseudomonas aeruginosa and association with clinical outcomes in patients: an observational study. J Antimicrob Chemother 77:793–798. doi:10.1093/jac/dkab461 34918135 PMC9097253

[34] Violet J, Smid J, Pielaat A, Sanders J-W, Avery SV. 2023. The influence of heteroresistance on minimum inhibitory concentration, investigated using weak-acid stress in food spoilage yeasts. Appl Environ Microbiol 89:e0012523. doi:10.1128/aem.00125-23 37255457 PMC10304792

[35] Tiseo G, Galfo V, Falcone M. 2023. What is the clinical significance of “heteroresistance” in nonfermenting Gram-negative strains? Curr Opin Infect Dis 36:555–563. doi:10.1097/QCO.0000000000000964 37729656 PMC10624410

[36] Doijad SP, Gisch N, Frantz R, Kumbhar BV, Falgenhauer J, Imirzalioglu C, Falgenhauer L, Mischnik A, Rupp J, Behnke M, et al.. 2023. Resolving colistin resistance and heteroresistance in Enterobacter species. Nat Commun 14:140. doi:10.1038/s41467-022-35717-0 36627272 PMC9832134

