# Supplementary material for: High-throughput clinical antimicrobial susceptibility testing and drug-resistant subpopulation detection in Gram-negative bacteria
Source: Microbiol Spectr. 2025 Jun 5;13(7):e00011-25. doi: 10.1128/spectrum.00011-25 (PMC12211026; doi:10.1128/spectrum.00011-25)
Supplement: Supplemental figures — Figures S1 to S3. [file spectrum.00011-25-s0001.docx]

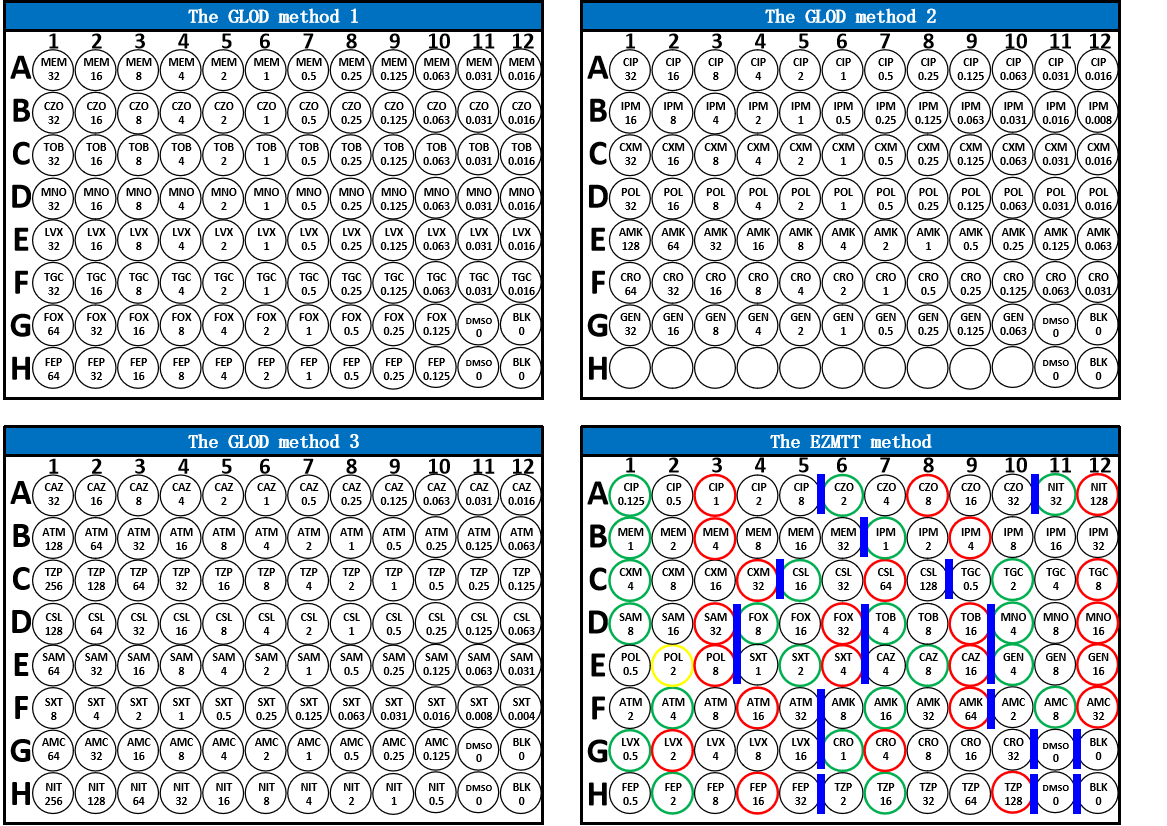


**Supplementary Fig. 1 The plate maps show the 2-fold dilution (GN#1, GN#2, GN#3) and the breakpoint based dilution (GN) plates.**


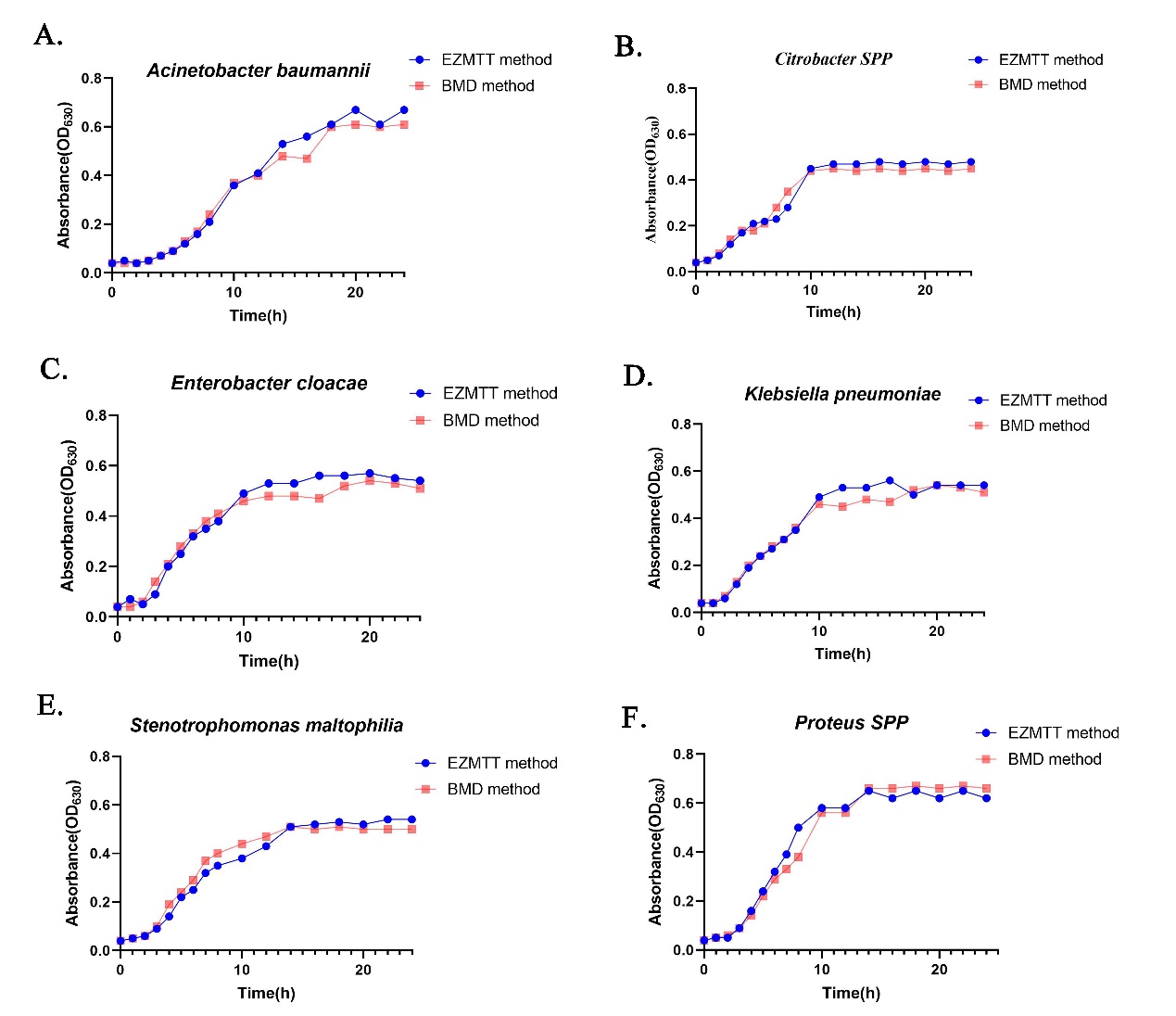


Supplementary Fig.2 **Growth curves of the *Enterobacter cloacae, K. pneumoniae, Citrobacter SPP., Proteus SPP, A. baumannii, and S. maltophilia* by** **the BMD method in the presence or absence of the EZMTT.** The growth of both bacterial strains was measured at 630 nm and essentially the same dose‒response curves were observed in the presence or absence of EZMTT , with a *P* value of >0.05.


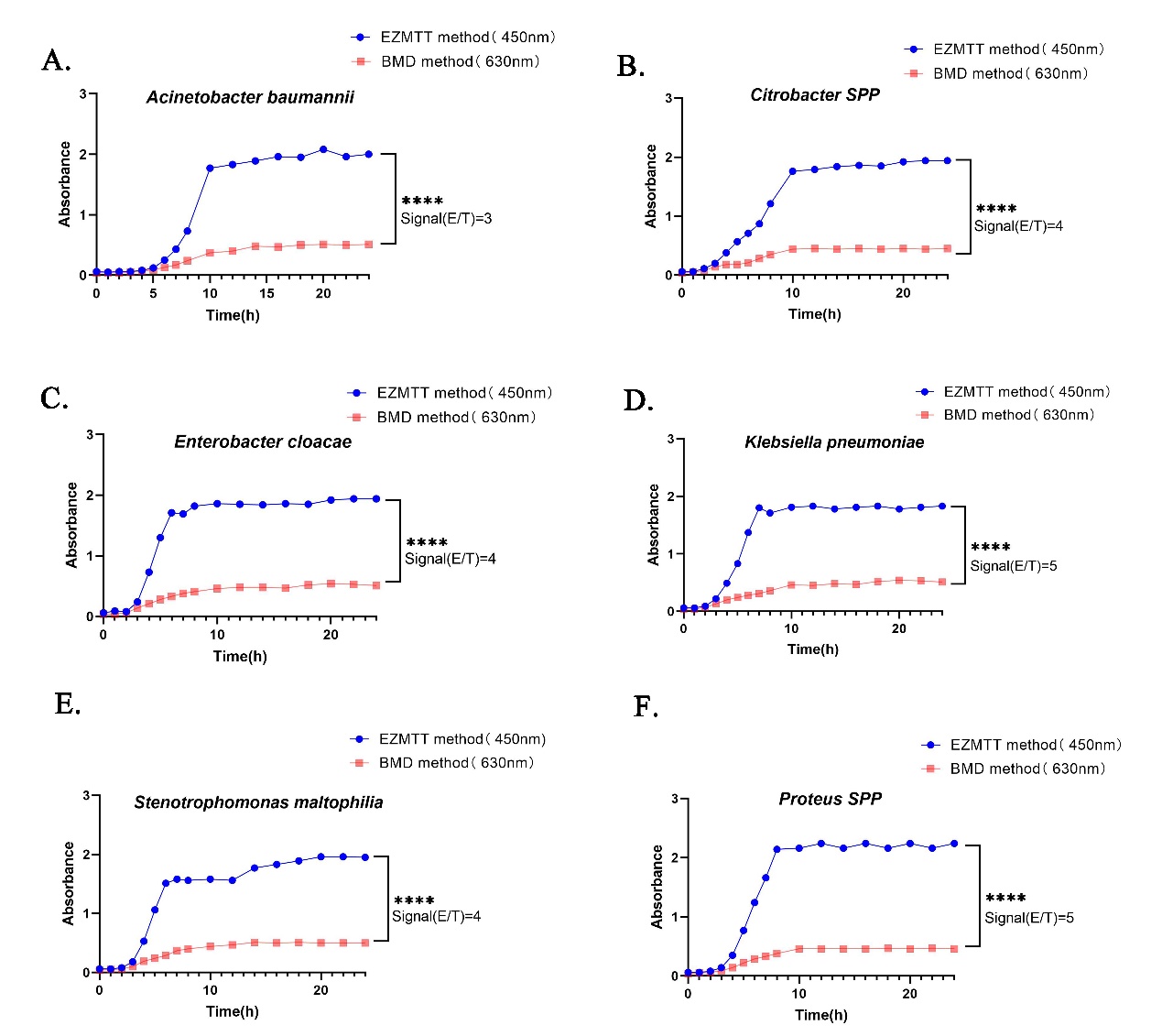


**Supplementary Fig. 3 Growth curves of the *Enterobacter cloacae, K. pneumoniae, Citrobacter SPP., Proteus SPP, A. baumannii, and S. maltophilia* by the BMD method in the presence or absence of the EZMTT.** The growth of both bacterial strains were measured at 450 nm or 630 nm, in the presence or absence of the EZMTT, respectively. Statistical analysis revealed a dramatic signal increase in the EZMTT method, with a *p* value of < 0.0001 (****).
