# Supplementary material for: High-throughput clinical antimicrobial susceptibility testing and drug-resistant subpopulation detection in Gram-negative bacteria
Source: Microbiol Spectr. 2025 Jun 5;13(7):e00011-25. doi: 10.1128/spectrum.00011-25 (PMC12211026; doi:10.1128/spectrum.00011-25)
Supplement: Table S1 — Comparison of AST results from both types of assay plates by the EZMTT assay with CLSI standards. [file spectrum.00011-25-s0002.docx]

**Supplementary Table 1. Comparison the AST results from both type of the assay plates by the EZMTT assay (450 nm) with the CLSI standards.**

**Note:** a. The breakpoint for IPM is for TIENAM, CFP for CSL; b. The MIC for POL in BMD assay in the presence or absence of the EZMTT are 4 fold lower than the CLSI values, which might come from the batch difference from manufactures; The MIC values were measured at 450nm, and if the MIC values reached the lower limit or upper limit of the dilutions, we marked the values with a ≤ or ≥ symbol.
